# Authorship: from credit to accountability: Reflections from the Editors’ Network

**DOI:** 10.1007/s12471-019-1273-y

**Published:** 2019-05-20

**Authors:** F. Alfonso, Editors’ Network, European Society of Cardiology (ESC) Task Force

**Affiliations:** Cardiology Department, Hospital Universitario de La Princesa, Instituto de Investigación sanitaria IIS-IP, Universidad Autónoma de Madrid, Madrid, Spain

**Keywords:** Editorial ethics, Scientific process, Authorship, Accountability, Scientific journals, Journals

## Abstract

The Editors’ Network of the European Society of Cardiology (ESC) provides a dynamic forum for editorial discussions and endorses the recommendations of the International Committee of Medical Journal Editors (ICMJE) to improve the scientific quality of biomedical journals. Authorship confers credit and important academic rewards. Recently, however, the ICMJE emphasised that authorship also requires responsibility and accountability. These issues are now covered by the new (fourth) criterion for authorship. Authors should agree to be accountable and ensure that questions regarding the accuracy and integrity of the entire work will be appropriately addressed. This review discusses the implications of this paradigm shift on authorship requirements with the aim of increasing awareness of good scientific and editorial practices.

The Editors’ Network of the European Society of Cardiology (ESC) is committed to fostering the implementation of high-quality editorial standards among ESC National Society Cardiovascular Journals (NSCJ) [1–6]. NSCJ play a major role in disseminating original scientific research worldwide, but also in education and harmonisation of clinical practice [2–6]. Promoting editorial excellence is paramount to increasing the scientific prestige of NSCJ [1–6]. In this regard, the Editors’ Network endorses the recommendations of the International Committee of Medical Journal Editors (ICMJE) [1]. The ICMJE continuously updates its document on uniform requirements (previously known as the Vancouver guidelines) for manuscripts submitted to biomedical journals. These include recommendations for the conduct, reporting, editing and publication of scholarly work. Notably, vexing ethical issues are gaining increasing editorial relevance [1].

Biomedical research relies on trust and transparency of the scientific process where authors remain centre stage [1, 7–9]. This review will discuss the new recommendations on authorship issued by the ICMJE [1, 10, 11] with the aim of providing further editorial insight to be progressively implemented by the NSCJ.

## New authorship requirements

In August 2013 an important revision of the ICMJE recommendations included a fourth criterion for authorship to emphasise each author’s responsibility to stand by the integrity of the entire work [1, 10, 11]. Classically, the ICMJE requirements for authorship included: (1) substantial contributions to the conception or design of the work or the acquisition, analysis, or interpretation of data for the work; (2) drafting the work or revising it critically for important intellectual content; *and,* (3) final approval of the version to be published. In the updated ICMJE requirements a new (fourth) criterion also should be met [1]. This novel requirement for authorship includes agreement to be accountable for all aspects of the work and ensuring that questions related to the accuracy or integrity of any part of the work are appropriately investigated and resolved [1]. The essence of this new requirement is that it helps to balance credit with responsibility [10]. With this revision the ICMJE emphasises that authorship is a serious commitment to accountability. Now all four conditions must be met by each individual author [1]. The addition of a fourth criterion was motivated by situations in which some authors were unable to, or refused to, respond to inquiries on potential scientific misconduct regarding certain aspects of the study or by denying any responsibility [1, 10–14]. Editors occasionally face reluctant authors who try to distance themselves from a conflictive publication and shift responsibilities elsewhere [11]. The main novel idea is to emphasise the responsibility of each author to stand for the integrity of the entire work. Each author of a scientific paper needs to understand the full scope of the work, know which co-authors are responsible for specific contributions and have confidence in co-authors’ ability and integrity [1, 10–14]. Should questions arise regarding any aspect of a study, the onus is on all authors to investigate and ensure resolution of the issue, which is then to be presented to the corresponding Editor [1, 10–14].

To better appraise this fourth criterion the precise meaning of responsibility and accountability should be revisited. *Responsibility* is defined as the moral obligation to ensure that a particular task is adequately performed [15, 16]. Accordingly, responsibility relates to tasks that have been assigned to an individual [15, 16]. By contrast, *accountability* denotes the duty to justify a given action to others and to respond for the results of that action [15, 16]. Therefore, accountability mainly relates to the awareness and assumption of the role of being the one to blame if things go wrong [15, 16]. Nevertheless, oftentimes responsibility is used interchangeably with accountability [15, 16].

Claiming that each individual author is held morally responsible in every case that misconduct is detected would appear unreasonable considering the complexity of current research. Rather, the fourth criterion suggests that each author must cooperate to clarify misconduct-related issues if the paper is called into question [1, 16].

### Research credits

Acceptance and publication of a scientific paper is always a cause of major celebration among authors [11]. Authorship provides prestige, credit and scientific recognition. Authorship has important academic, social and financial implications [1, 11]. Currently, authorship remains a major criterion for promotion and career advancement among scholars. Publication records are revised in depth for university tenures and job appointments. Total number of publications and citations remain currencies widely used to ascertain the academic value of individual investigators. In this regard, the ICMJE recommendations on authorship are intended to ensure that anybody who has made a ‘substantive’ intellectual contribution to a paper is given credit as an author [1].

### Potential problems derived from publication of research

Publication of a scientific paper usually marks the end of a research project and opens a time for discussion and criticism or acceptance by the scientific community [11]. Occasionally, the healthy scientific debate fuelled by the publication of the paper raises serious concerns. In rare cases, even the integrity of the research or published paper is brought into question [11]. In these situations authors may try to escape from the embarrassment of publishing a scientifically flawed study. This explains why the new fourth criterion is so pertinent to address issues related to scientific misconduct. Should irregularities be confirmed, editors must report to the authors’ academic institution and, eventually, to the readers, with expressions of concern, or, in the worst-case scenario, with a retraction of the published paper [1].

## Considerations on classical authorship criteria

Any researcher listed as an author should have made a ‘substantive’ intellectual contribution to the study and be prepared to take public responsibility for the work, ensure its accuracy, and be able to identify his/her contribution to the study [1]. However, a problem with the definition of authorship involves the subjectivity in what constitutes a ‘*substantial*’ contribution to the research or the manuscript. In fact, the precise threshold of involvement required to qualify for authorship remains unclear. As the real problem lies in defining what represents a ‘substantial’ contribution, means to quantify the actual work performed by individual authors have been proposed. In this regard it has been suggested [17] that substantial contribution to a publication consists of an important intellectual contribution without which a part of the work or even the entire work could not have been completed or the manuscript could not have been written [17].

According to the ICMJE [1] persons who *do not* qualify as an author include those who ‘*only*’ provide: (1) recruitment of patients to a trial, (2) general data collection, (3) obtaining samples for a study, (4) acquisition of funding, (5) general supervision of the research group by the department chairperson. Conversely, persons who significantly contributed to the paper but do not meet the four criteria for authorship should be listed in the acknowledgement section after obtaining their consent.

## Publishing individual contributions

The ICMJE authorship guidance is intentionally broad and open to accommodate the diversity of scientific research and allow space for the specific editorial policies of individual journals [1]. However, many have requested a more structured authorship framework to improve consistency and clarity in authorship requirements. The best means to present the relationship between authorship and intellectual involvement in research remains an issue of ongoing debate. Currently, the ICMJE does not mandate that all authors communicate exactly what ‘contributions’ qualify them to be an author [1]. However, unless authorship reflects to what extent individual researchers have been intellectually involved in the work it will remain misleading regarding relative research merits. Honesty and openness in attribution ensures fairness in credit. Many editors argue that authorship criteria should be revised to request a contribution declaration, in order to fully capture deserving authorship and credit. Accordingly, to promote transparency and remove ambiguity on specific contributions, editors are now strongly encouraged to develop and implement contributorship policies in their journals [1]. As discussed, however, the question regarding the quality and quantity of contribution required to qualify an individual for authorship remains unresolved [1]. An interesting proposal in this regard suggests including contributorship badges. These badges are designed to fully capture the different types of collaboration in the submitted work that, otherwise, will be difficult to recognise with traditional credentials. Listing of contributors allows a more accurate and granular assessment of credit. In addition, this strategy provides additional insight on contributor-adjusted productivity [18]. Ideally, each ICMJE criterion should have at least one badge. Each badge includes a list of authors making a contribution to that specific role [18–20]. Others have proposed the value of assigning a numerical value to better evaluate the degree of relative contributions and, eventually, to create a contribution-specific index for each author to better assess research productivity [18–20].

Detailing authors’ contributions informs the readers of the nature of the individual work and avoids diluting credits by precisely allocating merits. In multi-authored papers it is particularly important that authors state the specific role they played in the research. Each research project represents a significant amount of effort and, on average, the larger the number of authors the smaller percentage of effort for a given author. Other forms of contributions, not fulfilling criteria for authorship, may be recognised in the acknowledgement section or by listing these people as collaborators. This is an important issue considering the ever-increasing number of authors seen in recent publications that represents a paradigm shift resulting from team-work research [18–24]. Contributors credited as authors should take full responsibility and remain accountable for what is published [1, 18]. In this regard, contribution-adjusted credits can be further weighted by other factors to derive more effective parameters for measuring research productivity. Currently, every co-author receives the exact amount of citation credit regardless of their contribution. Therefore, an ‘author matrix’ (including participation in ideas, work, writing and stewardship) has been proposed to ‘quantify’ individual contributions and roles in multi-authored papers [18–24].

## By-line location and hierarchy

There is no adequate guidance for author sequence in the by-line. In fact, practices to clarify the relative merit of the different co-authors in a manuscript vary significantly among scientific disciplines [18–22]. For biomedical journals, the first author is the most important position, followed by the last author and then the second author. The first author is reserved for the person who made the largest contribution (investing most time in the project), usually the author who wrote the first draft of the paper. Then the sequence of authors tends to represent progressively lesser contributions [18]. Following this approach, where the sequence determines credit, the last author receives the least. Accordingly, the last position might be considered as a rather generous option. Actually, the last position is currently considered as very important in biomedical research and, in fact, it is frequently associated with the corresponding author or the guarantor of the entire work [18]. However, many argue that senior scientists should grab the pen (keyboard) more often, as writing remains essential for advancement in knowledge [19]. Senior authors have the responsibility to promote the academic career of new-generation scientists.

Many journals allow authors to declare that two or more individuals have made an ‘equal contribution’ to the research [21, 25–28]. In the last decade the percentage of articles with equal contribution statements has increased dramatically both in basic sciences and medical journals [25]. Notably, the designation of ‘joint first authors’ should be based on the quality and quantity of the work [21, 25–28]. Thus the “contributed equally” designation should be reserved to honestly reflect similar scientific contributions and not to inflate a curriculum vitae [21, 25–28]. Interestingly, the practice of listing two individuals as ‘joint last author’ is used less frequently but is steadily increasing. These publications should include a footnote clearly indicating that both authors contributed equally to the work [21, 25–28].

The corresponding author takes primary responsibility for communication with the journal during the submission, peer-review, publication and post-publication periods [1]. Currently, most journals require contact e‑mail addresses from all listed authors who then will be contacted to inform them that the corresponding author submitted the paper. This ensures that they are aware that the paper has been submitted in their name. The systematic implementation of this electronic warning system paves the way to guarantee that the third authorship criterion has been met. Therefore, the policy now may be considered as a mere administrative requirement similar to signing a copyright transfer form.

The ‘guarantor’ of the study may be different from the first or corresponding author and frequently is the principal investigator or most senior person in the group. The guarantor takes full responsibility for the integrity of the work as a whole from inception to the published paper. Accordingly, the guarantor must be fully prepared to defend all parts of the research project and final manuscript. Guarantors vouching for the integrity of the entire work are of special value for multi-author articles particularly when many institutions are involved. All authors should also disclose potential conflicts of interest [1, 5]. The ICMJE uniform conflict of interest disclosure has been recently updated and all authors should complete the corresponding standardised individual electronic document [1, 5]. In particular, authors of sponsored studies should indicate that they had full access to the data and take complete responsibility for the accuracy and integrity of the analysis. This is important as the roles and interests of different stakeholders may remain elusive or misleading in this type of study [1].

The subjectivity and emotionality of authorship may explain why disputes among investigators are not uncommon. Authorship disputes amongst research teams should be avoided by deciding roles and responsibilities beforehand. Ideally, the order of the authors should be collectively decided by the research team at the onset of the project [29]. Then, the definitive order should be revised when the work is completed, taking into account the actual level of individual contributions [17]. Editors are unable to judge whether authors have met the authorship criteria. The COPE (Committee on Publication Ethics; www.publicationethics.org) guidelines are useful to solve publication disputes [9]. Editors should seek explanations and the signed agreement of all authors if there is a request for a change in the author list [1].

## Multi-authored articles

Scientific collaboration has become increasingly important because the complexity of modern research involves different competencies [16]. Moreover, a large number of patients and centres may be required to adequately address clinically relevant questions [16]. In addition, multidisciplinary research groups offer the opportunity of cross-pollination [16]. Therefore, team-work is currently commonplace in biomedical research. Co-authorship is the most tangible result of multilateral scientific collaboration. Group (corporate) authorship has become increasingly common with variations in how individual authors and research group names are listed in the by-line. Notably, citation impact is greater in papers with multiple authors coming from international cooperation. The problem of inflating publication and citation records of authors participating in multicentre studies has been a cause for concern [18]. This is due, at least in part, to collaboration-induced self-citation [30]. Salami publications, or least publishable units strategies, are initiatives that inflate the number of publications on the same research project by dividing the work (that could have been presented in a single main paper) into smaller component parts, then publishing them as several different articles. Such strategies may be detected in some multicentre studies [30]. The use of co-author-adjusted citation indexes has been suggested to account for this phenomenon [30].

There is evidence that the number of co-authors per paper in medical literature has increased exponentially over time [22, 31]. The reason for this increase is probably multifactorial and includes increasing complexity of research, as discussed, but also author inflation. Inappropriate authorship is not ethical and eventually leads to diminishing the value of authorship, generating a situation where undeserved co-authors cannot take responsibility for the research [22, 31]. Interestingly, the correlation between research quality and number of authors is poor, suggesting that the component of author inflation plays a greater role than that of research complexity [31].

Until now the number of authors in the by-line was not considered in the evaluation of the relative academic merit of individual authors [3]. However, as a research project involves a defined amount of work, the larger the number of authors in a paper the less merit any given author deserves. Major efforts are made by some individuals whereas others contribute significantly less. The credit received by people doing the work becomes diluted by the inclusion of many authors with minor, if any, contributions. Eventually this ‘free lunch’ strategy undermines the value of being named on a scientific paper [32].

Authorship guidelines should be updated to adapt to the growing trend of collaborative research. The larger the number of authors the more opportunities for contentious arguments and disputes. Every author of a ‘group authorship’ work must meet the four criteria for authorship. Otherwise they should be identified just as investigators or collaborators rather than authors [1]. Given the complexity and multiple tasks involved in current research it is clear that most authors cannot participate in every aspect of the work. Accordingly, specific responsibilities should be tied to different research roles. Authors should refrain from collaborating with colleagues whose quality or integrity may inspire concerns [1]. Last, but not least, with a growing number of authors it is increasingly difficult to identify those who may be held morally responsible should scientific misconduct be detected [22, 31]. Holding everybody responsible is unfair to the researchers that are not guilty of misconduct.

## Breaches in authorship: from ghost to guest authors

Breaches in authorship are a form of deception. Guest or gift (honorary) and ghost (hidden) authors represent a form of authorship abuse that should not be permitted [33–37]. Ghost authorship is omitting authors that have made relevant contributions to a paper. Ghost authors provide contributions to a manuscript that do merit authorship but, for different reasons, are not included in the author by-line. Some ghost authors may have major conflicts of interest or are paid by a commercial sponsor. This should be differentiated from ghost writing. Ghost writers are writing contributors to a manuscript that do not fulfil authorship criteria, but their contributions are not disclosed in the acknowledgements [17, 36]*.* Ghost writing is also an unethical practice as it keeps hidden the involvement in the manuscript. The concern is that writers hired by industry might influence the content of the publication or hide unwelcome results, which introduces potential bias that is obscured when relevant academic guest authors are accredited with authorship [17]. Professional medical writers should follow ethical publication practices and should openly disclose their involvement in the acknowledgement section [36].

The inclusion of individuals with minimal or no input reflects ‘loose authorship’ practices [33–37]. Guest, gift or honorary authorship is defined as co-authorship awarded to people who do not meet the authorship criteria and have not contributed substantially to taking public responsibility for the work [1]. This may be offered in the belief that the prestige of a scientifically respected person will increase the likelihood of publication or the impact of the work [29]. Oftentimes, a well-known academic senior name is used to conceal ghost authors with industry-related conflicts of interest [29]. Both the gift author and the remaining co-authors may benefit from this practice (a win-win situation) that, nevertheless, remains unethical. The increased pressure to publish among scholars seeking promotion and career advancement (the “publish or perish” culture) may also help to explain these practices. This pressure explains why some researchers accept ‘gift’ authorship in papers to which they have not contributed intellectually. This abuse in authorship devalues the merit of being named as an author in a scientific paper. As previously discussed, quantitative contribution helps to prevent granting undeserved credits to guest authors who take away well-deserved credits from the authors who actually did the work [37–40].

Studies suggest that breaches of authorship guidelines are frequent. In a recent survey one-third of authors believed that they had been excluded from deserved authorship and a similar number declared that they had experienced pressure to include undeserved authors in their papers [20]. Another recent study of journals included in the Journals Citation Reports database suggested that 85% of them included in their policy guidance the requirement that authors should be accountable for the research as a whole, 32% explicitly prohibited guest or ghost authorship, but only 5% required authors to describe their individual contributions [25].

## Appendix

**Table 1:** Editors in Chief and their journals

Alban Dibra	Editor in Chief Revista Shqiptare e Kardiologjisë
Albert Varga	Editor in Chief Cardiologia Hungarica
Alexander Mrochek	Editor in Chief Cardiology in Belarus
Alfonso Buendia Hernandez	Editor in Chief Archivos de Cardiologia de Mexico
Andreas Flammer/François Mach	Editor in Chief Cardiovascular Medicine
Anetta Undas	Editor in Chief Kardiologia Polska
Anton Doubell	Editor in Chief SAHeart
Ariel Cohen	Editor in Chief Archives of Cardiovascular Diseases
Carlos Eduardo Rochitte	Editor in Chief Arquivos Brasileiros de Cardiologia
Carmen Ginghina	Editor in Chief Romanian Journal of Cardiology
Çetin Erol	Editor in Chief Anatolian Journal of Cardiology
Chu-Pak Lau	Editor in Chief Journal of the Hong Kong Colleage of Cardiology
Claes Held	Editor in Chief Svensk Cardiologi
Dario Echeverri	Editor in Chief Revista Colombiana de Cardiologia
Dilek Ural	Editor in Chief Archives of the Turkish Society of Cardiology
Dimitris Tousoulis	Editor in Chief Hellenic Journal of Cardiology
Eckart Fleck	Editor in Chief Der Kardiologe
Evgeny Shlyakhto	Editor in Chief Russian Journal of Cardiology
Faiq Guliyev	Editor in Chief Azerbaijan Journal of Cardiology
Faouzi Addad	Editor in Chief Revue Tunisienne de Cardiologie
Gerd Heusch	Editor in Chief Basic Research in Cardiology
Germanas Marinskis	Editor in Chief Seminars in Cardiovascular Medicine
Giuseppe Di Pasquale	Editor in Chief Giornale Italiano di Cardiologia
Ignacio Ferreira-González	Editor in Chief Revista Española de Cardiología
Ismael Guzman Melgar	Editor in Chief Revista Guatemalteca de cardiologia
Izetbegovic	Editor in Chief Medicinski Zurnal
Jan Piek	Editor in Chief Netherlands Heart Journal
Jean-Jacques Monsuez	Editor in Chief Archives des maladies du cœur et des vaisseaux-Pratique
Kanat Kabdrakhmanov	Editor in Chief Journal Terapevticheskiy vestnic
Kjell Nikus	Editor in Chief Sydänääni (Heart Beat)
Kurt Huber	Editor in Chief Austrian Journal of Cardiology
Laila Haddour	Editor in Chief Revue Marocaine de Cardiologie
Lino Goncalves	Editor in Chief Revista Portuguesa de Cardiologia
Livio Dei Cas	Editor in Chief Journal of Cardiovascular Medicine
Luc Pierard	Editor in Chief Acta Cardiologica
Mahmoud Hassanein	Editor in Chief The Egyptian Heart Journal
Mamanti Rogava	Editor in Chief Cardiology and Internal Medicine (Georgian International Society of Cardiomyopathy)
María del Pilar Aguilar Passano	Editor in Chief Revista Uruguaya de Cardiologia
Mario Ivanusa	Editor in Chief Cardiologia Croatica
Michael Aschermann	Editor in Chief Cor et Vasa
Michael Boehm	Editor in Chief Clinical Research in Cardiology
Mikael Sander	Editor in Chief Cardiologisk Forum
Nuray Enç	Editor in Chief Kardiyovaskuler Hemsirelik Dergisi
Olaf Rødevand	Editor in Chief Hjerteforum
Parounak Zelveian	Editor in Chief Armenian Journal of Cardiology
Plamen Gatzov	Editor in Chief Bulgarian Journal of Cardiology
Robert Hatala	Editor in Chief Cardiology Letters
Slobodan Obradović	Editor in Chief Heart and Blood Vessels
Tzung-Dau Wang	Editor in Chief Acta Cardiologica Sinica
Ulrike Fortmüller	Editor in Chief Cardio News
Valentyn Shumakov	Editor in Chief Ukrainian Journal of Cardiology
Yangsoo Jang	Editor in Chief Korean Circulation Journal
Zlatko Fras	Editor in Chief Slovenska kardiologija
